# 

**Published:** 1893-07

**Authors:** 


### LITERARY.

UNCLE TOM'S CABIN has certainly "broke loose!" The copyright on this most famous of American novels, by Mrs. Stowe, has recently expired, which frees its publication from the monopoly of the high-priced publishers and though in anticipation of this fact they have within a few months greatly reduced its price; now that it is really "unchained,"the consequences are something surprising. John B. Alden, Publisher, of New York, issues several editions selling them only *direct*, not through agents or booksellers; one in good type, paper covers for 5 cts sent post paid, or the same bound in cloth for 10 cts, with postage 7 cts extra; also an excellent large type edition on fine paper handsomely bound in cloth for the price of 25 cents, postage 10 cents. Surely a copy of Uncle Tom's Cabin will soon be found in every home where it is not already. Mr. Alden sends a thirty-two page pamphlet describing many of his publications free or a catalogue of one hundred and twenty eight pages of choice books, a veritable literary gold mine for book lovers, for two cents. Address John B. Alden, Publisher, 57 Rose St, New York.

PHYSICAL EDUCATION, for June (published at Springfield, Mass. $1.00 per year), is a number of unusual interest. An article on the "Gymnastic Treatment of the Feeble Minded," shows how, through the connection of exercise of muscle, it is possible to exercise and thus develop those parts of the brain that have to do with muscular contraction, and that thus, as well as in other ways described, the brain can be stimulated to develop.

That this is not mere theory is shown by the fact that Dr. Gulick, the writer, is describing simply what he himself has done, giving the reasons for it.

A continued article by Dr. Hitchcock, of Cornell, and R. F. Nelligan, of Amherst is on "Wrestling," and consists mainly of illustrations taken from life, showing the various holds and breaks in the catch-as-catch-can style.

Besides these and major articles there is much of minor interest.

It is a question worthy of some consideration as to the best form in which to reproduce and preserve such features of the World's Fair buildings and exhibits as are best worth preserving. To gather, shelter and properly display in so great profusion the results of the achievements of civilization, at a cost of fifty or sixty millions of dollars only in a brief period, when the buildings are taken down and the exhibits removed, to lose it all, to have preserved no proper representation or description of the brilliant though fleeting spectacle, would be a serious mistake. Obviously, there is but one way in which this can be done, and that is in the form of a book, print and pictures in about equal parts, neither so large as to be cumbersome, and yet large enough to do the subject full justice.

Such is the plan of THE BOOK OF THE FAIR, to be issued in twenty-five parts of forty imperial folio pages each, by The Bancroft Company, Auditorium Building, Chicago. The most thorough and elaborate preparations were made by these publishers to produce what should be in the highest and best sense a work of art and of utility, a book which should be at once beautiful and useful. The best processes were adopted and the best artists secured to illustrate the text, which is by Hubert Howe Bancroft. We have here, then, the history and description of the entire Exposition by an author of known character and repute, aided and adorned by the most beautiful pictures that can be produced. It is safe to say that in no other form can the great Exposition be so well presented and preserved.

THE HYGIENE OF THE SICK ROOM. A book for nurses and others, Being a brief consideration of Asepsis, Antisepsis, Disinfection, Bacteriology, Immunity, Heating and Ventilation, and kindred subjects, for the use of nurses and other intelligent women. By William Buckingham Canfield, A. M., M. D., Lecturer of Clinical Medicines and Chief of Chest Clinic, University of Maryland. Philanelphia, P. Blakiston, Son & Co., 1892. Pp. 247, Cloth $1.50.

Every home should obtain this book; a brief, terse and exhaustive treatise on nursing; invaluable to the household library.

BLUE AND GRAY, for June, is a rich number "The New Republic" is admirably treated by Edwin Lewis Suter.

Charles F. Currie writes an interesting article on "New Jersey Blue honors Alabama Gray," profusely illustrated.

Louisa Howard Bruce writes tersely on "At War with Love," illustrated.

A popular feature of the magazine is the Youth's department. Popular price $2.50 per year.

The west comes to the front with an attractive and valuable publication the COLORADO MAGAZINE. About the size of the Century, price $2.50 per year.

The June number contains an interesting article entitled "A Peck of Diamonds," by Frank G. Carpenter, the popular newspaper correspondent. The Naval Rendezvous and Review, is richly treated with illustrations by Lieut. Willoughby Walker.

A short story entitled "Brace Magruder," by Lewis B. France, claims a notice. ' The Progress of Medical Science," carefully and thoroughly reviewed by H. T. Goodwin, M. D., Ass't Surgeon U. S. Marine Hospital Service.

The CENTURY is full of good things. It opens with an illustrated paper from the diary of Lieut. William Henn, under the title "Caught on a Lee shore." Writing to Rosini," by William Henry Bishop, is a pure, simple thoughtful story of absorbing interest. Frederic Remingtons name has become a world wide one especially through his wonderful and interesting portrayal of life on the western plains. "In Cowboy Land," is his latest and is well illustrated. The number is a rich one.

THE TOURIST, for June, a monthly publication, illustrated, published at Utica, by F. G. Barry, is on our review table and is at once attractive by its neat typographical appearance. Well edited, and appropiately illustrated, with contents worthy its unique name, gives it a rank among the leading periodicals of its kind in the country. $1.00 per year, or clubbed with Journal, for $1.50.

THE COSMOPOLITAN, for June is as usual, attractive. The introductory article is of local interest The City of Brooklyn, by Murat Halstead, and is handsomely illustrated. "The Rise and Decline of the Hawaiian Monarchy," is a rich article, written by Herbert H. Gowen. "Omega, the Last Days of the World," is continued by Camille Flammarion.

We heartily welcome the first number of the Popular Health Magazine published in Washington, D. C.

Forty-four pages one dollar per year. The publishers of the Journal congratulate the publishers of the magazine upon the neat attractive and well edited periodical.

We have received the first number of the Electro-Light Engraving Co's monthly which the Company announces will appear regularly. Eight pages in size and it portrays beautifully the superiority of their fine work along the line of engraving of all descriptions.

CREMATION AND ITS IMPORTANCE to CHOLERA is the title of an abstract of a discussion at a meeting of the Northwestern Medical and Surgical Society of New York, last January, by Robert Newman, M. D., of 68 West 36th st., New York. It is a ten page pamphlet and it ought to have a thorough distribution.

